# Multiparametric ultrasound-based assessment of overt hyperthyroid diffuse thyroid disease

**DOI:** 10.3389/fendo.2023.1300447

**Published:** 2023-12-18

**Authors:** Dana Stoian, Andreea Borlea, Luciana Moisa-Luca, Corina Paul

**Affiliations:** ^1^ Discipline of Endocrinology, Second Department of Internal Medicine, University of Medicine and Pharmacy “Victor Babes”, Timisoara, Romania; ^2^ Center for Molecular Research in Nephrology and Vascular Disease, “Victor Babes” University of Medicine and Pharmacy, Timisoara, Romania; ^3^ Department of Pediatrics, University of Medicine and Pharmacy “Victor Babes”, Timisoara, Romania

**Keywords:** thyroid ultrasound, multiparametric ultrasound, 2D SWE, hyperthyroidism, diffuse thyroid disease

## Abstract

**Introduction:**

Hyperthyroidismis a prevalent condition affecting global populations, with an overall prevalence of 1.2%. Our research aimed to establish a systematic diagnostic approach using multiparametric ultrasound (MPUS) to diagnose hyperthyroid diffuse thyroid disease (DTD).

**Methods:**

We conducted a retrospective study from June 2021 to June 2023 at a specialized endocrinology center in Timisoara, Romania, enrolling subjects presenting with clinical hyperthyroidism. Using the Mach 30 Aixplorer ultrasound equipment, evaluations were performed initially in B-mode US, followed by Color Doppler and Spectral Doppler measurements, and finally, 2D Shear wave elastography (SWE).

**Results:**

From the 218 patients analyzed, the diagnosis of DTD with hyperthyroidism was confirmed through biochemical assessment, subgrouping various pathologies such as subacute thyroiditis, Graves’ disease, painless thyroiditis, Hashimoto’s thyroiditis, iatrogenic, as well as healthy controls. In the first step, B-mode hypoechogenicity had an AUC of 0.951 for DTD detection. In the second step, the peak systolic velocity differentiated Graves’ disease with a median of 42.4 cm/s and an AUC of 1. Lastly, the third step consisted of SWE evaluation, revealing a mean elasticity index in the SAT subgroup significantly higher from other subgroups (p<0.001) with an AUC of 1.

**Conclusion:**

Our study offers a step-by-step evaluation algorithm for DTD diagnosis, with a very good overall diagnostic performance (AUC of 0.946).

## 1 Introduction

Hyperthyroidism is a common condition with potentially devastating health consequences that affect all populations worldwide (1), with an overall prevalence of 1,2%; 0,5% overt forms and 0,75% subclinical forms (2). The annual incidence in Europe stands at 51/100.000 cases (3). Autoimmune causes, hyperfunctional nodular hyperplasia disease, or excessive use of thyroxine or human chorionic gonadotrophic hormone, are the most frequent causes of hyperthyroidism (4). Factors like age, smoking status, genetic susceptibility, ethnicity, exposure to endocrine disruptors, and innovative therapies such as immune checkpoint inhibitors also influence thyroid disease epidemiology. Mean daily iodine intake is a major risk factor for thyroid disease (1). Besides overproduction of thyroid hormones, there are different types of thyroiditis, where exposure to inflammation, post viral response, use of lithium, interferon alfa, interleukin -2, amiodarone and immunotherapy or triggered by childbirth causes a destruction of thyroid follicles (5).

The clinical diagnostic of hyperthyroidism is straightforward, but the question to be answered in front of any case of hyperthyroidism despite overt or subclinical stage of disease, is whether there is a case with an overproduction of thyroid hormones when antithyroid medication is considered (2), respectively there is a destructive case, in which the control of inflammation/aggression is mandatory. Conventional, thyroid scintigraphy (TS) is used, to make the differential diagnostic (6). Yet, the rise of subacute thyroiditis (SAT) during the Covid period (7, 8), highlighted the need to limit unnecessary evaluations and reduce hospital pressures, especially since atypical forms of thyroiditis were observed after the viral infection (9).

Ultrasound remains the primary morphological tool for thyroid disease diagnosis, both nodular and diffuse. Multiparametric ultrasound evaluation is used in the differential diagnostic of focal liver (10) and pancreatic lesions, breast (11, 12), thyroid (13–17), cervical lymph nodes (18)or testicular (19–21) nodular disease. Combining the information from gray scale US, doppler, elastography and contrast enhanced ultrasound do offer valuable information also in diffuse diseases such as salivary gland (22) or liver (23, 24).

In B-mode ultrasound, criteria like altered echogenicity, coarse echo structure, lobated margins or anteroposterior lobar diameter higher than 2 cm suggest diffuse thyroid disease (DTD) with high sensitivity and specificity (87.6% and 92.1%) (25–27).

Despite ongoing debates about its proper application and interpretation (28), color doppler (CD) is described as a helpful technique in the diagnosis of Graves’ disease (GD) from destructive thyroiditis (29, 30), although initial phase of Hashimoto thyroiditis (HT) overlaps increased diffuse vascular pattern (31). Even the use of Schulz visual scale does not significantly increase the diagnostic accuracy of CD (32). The quantitative appraisal of the blood flow, by measuring the thyroid artery Peak Systolic Velocity (PSV) has improved in accuracy and dependability, with excellent consistency in the differential diagnostic of Graves’ versus other forms of thyroiditis (29), since the PSV shows a direct correlation with the radioactive iodine uptake, measured at 3 and also 24-hour time span (33). The cutoff value for differentiating GD from thyroiditis is 40-50 cm/sec (29, 33, 34).

Extrapolating the excellent results observed in the diffuse chronic liver disease, elastography was used in the differential diagnosis of DTD from normal thyroid. Both strain and 2D-shear wave elastography (2D-SWE) demonstrated increased stiffness in autoimmune thyroid disease compared with normal thyroid (35, 36), both in adult (37) and pediatric populations (38, 39).

Considering all the information provided, we aim to establish a step-by-step diagnostic procedure, utilizing universal MPUS for the positive diagnosis of DTD.

## 2 Materials and methods

### 2.1 Patients

A retrospective monocentric investigation was carried out between June 2021 - June 2023. A total of 168 subjects with clinical hyperthyroidism at initial diagnosis before any treatment was initiated and 50 controls were enrolled, which presented for evaluation in the Ultrasound Department of a specialized endocrinology center in Timisoara, Romania. Clinical hyperthyroidism was defined as low thyroid stimulating hormone (TSH) values and free thyroid hormones above the reference range, at the moment of evaluation confirmed by biochemical assays performed within one week. Patients with hyperthyroidism caused by toxic adenoma or toxic multinodular goiter were excluded (see **Supplementary Figure 1**). Before joining the study, every participant gave their written informed consent. The research adhered to the standards set by the Declaration of Helsinki, as revised in 2000 in Edinburgh, and received approval from the Local Ethics Committee of our Institution (nr. 235/2021).

### 2.2 Ultrasound evaluation

All cases were evaluated with multiparametric US (MPUS), using a Mach 30 Aixplorer ultrasound equipment (Hologic Inc, Production year 2020) and a 8-15 MHz multifrequency linear probe. The patient was positioned supine with the neck extended throughout the ultrasound examination, and coupling gel was used to seal the transducer’s contact with the neck skin. All evaluations were carried out by an US operator (D.S.) with more than 5 years of thyroid 2D SWE expertise.

The following ultrasound techniques were universally applied:

- Initially in B-mode US, the thyroid volume and echogenicity were evaluated. The thyroid volume was calculated in each case by measuring for each lobe the anteroposterior, transverse, and longitudinal diameter and calculated by the machine using the ellipsoid volume formula; the echogenicity was qualitatively assessed by the US operator as isoechoic (0), mildly (1) or intensely hypoechoic (2) (compared to salivary gland aspect) (40);
- Color Doppler was performed for both lobes, using Schulz qualitative scale (32) with gradings from 0 to III. Pattern 0 was described when blood flow is found only in the external thyroid arteries, pattern I describes a slight rise in blood flow within the thyroid tissue, pattern II is described when noticeably enhanced flow is evident, distributed uniformly throughout, respectively pattern III when significantly increased blood flow is seen in Doppler assessment, uniformly spread, also including the picture known as “thyroid inferno”;
- Spectral Doppler with measuring the systolic speed in the superior thyroid arteries (PSV), on both sides. The Doppler angle was kept at or below 60° and the correction angle was adjusted parallel to the direction of flow (29, 33). Three measurements were made for each side. The values were measured in cm/sec.
- 2D Shear wave elastography was performed in the same evaluation. For image acquisition and tissue elasticity quantification, the 2D ShearWave (2D-SWE PLUS™) mode was enabled. In addition to displaying the elasticity color-map (blue-green-yellow-red), as estimated by the different shear wave speed, the machine also offers a numerical parameter, the Young’s modulus or elasticity index (EI), expressed in kilopascal (kPa). The 2D-SWE PLUS map is displayed alongside the B mode image, with a maximum set at 100 kPa for thyroid evaluation. For the image acquisition, the thyroid lobe was included in the SWE box, and the region of interest (QBox™) was put in an area where the color-map was fully coloured and free of artefacts. After a 5-second image stabilisation and with a steady color-coded map displayed on the device’s screen, 3 measurements were taken in each thyroid lobe, avoiding artifacts. Measurements that deviated significantly from the median of the three measurements for a specific thyroid lobe were flagged as potential outliers. These outliers were subject to further review and, if necessary, verification.
- The Qbox’s diameter was predetermined to be between 5 and 10 mm. The following parameters were recorded: the minimum EI (Emin); the maximum EI (Emax); the mean EI (Emean) and the standard deviation (SD).

### 2.3 DTD Diagnosis

In all cases functional thyroid tests were determined: free-thyroxine (FT4) (method immunochemistry with enzyme chemiluminescence immunoassay - ECLIA; reference values 0.93–1.7 ng/dL), free triiodothyronine (FT3) (method ECLIA; reference values 2.21-4.43 pg/ml), thyroid-stimulating hormone (TSH) (method ECLIA; reference values 0.27–4.20 mIU/L). Clinical hyperthyroidism at onset was present in all the included cases. We finally included in the study patients that had performed the golden standard examination for confirming the diagnosis in each type of pathology and all patients included in the study were at the onset of thyroid disease.

The first subgroup consisted of patients with subacute thyroiditis which was suspected by clinical examination (anterior neck pain and tenderness) and by the presence of inflammation markers: increased C-reactive protein (CRP) and or erythrocyte sedimentation rate (ESR) and confirmed by technetium scintigraphy showing low or absent thyroid uptake. The second group was composed of patients with Graves’ disease which was confirmed by an increased titer of TSH-receptor antibodies (TRAb) (clinical decision limit <1.75 IU/L normal range; method ECLIA). The iatrogenic group included patients that were treated with amiodarone, radiotherapy, and immunotherapy, with the exclusion of other causes. Hashitoxicosis was diagnosed in hyperthyroid patients with negative TRAb and positive anti-thyroid antibodies: anti-thyroid peroxidase antibodies (ATPO) (method microparticle-based chemiluminescence immunochemistry - CMIA; normal range <34 IU/mL) and/or anti-thyroglobulin (ATG) antibodies (method ECLIA; normal range <115 IU/mL). The diagnosis of painless thyroiditis was established in patients that were not included in any of the above-mentioned categories, with or without positive antithyroid antibodies. All controls had normal thyroid function tests and negative antithyroid antibodies. We excluded from our study patients that were already diagnosed with DTDs and were already taking antithyroid medication or levothyroxine supplementation, patients that did not have the golden standard confirmation of the disease, woman that were pregnant or less than 6 months post-partum and cases of exogeneous hyperthyroidism.

### 2.4 Statistical analysis

The statistical analysis was performed using MedCalc v12.5 software, developed by MedCalc Software in Belgium. Descriptive statistics were employed to characterize the demographic and clinical data, as well as the ultrasound findings. The assessment of normality for numerical variables was conducted using the D’Agostino-Pearson test. The numerical variables that exhibited normal distributions were characterized by their mean and standard deviation. On the other hand, variables with non-normal distribution were represented by their median and interquartile range (IQR) spanning from the 25th to the 75th percentile. Figures and percentages were utilized to depict qualitative characteristics. The Mann-Whitney U-test was performed to analyze non-parametric variables, whereas parametric variables were assessed using the parametric t-test. The box plots allowed for the effective depiction and comparison of median values, enabling a thorough visual description of the central tendency as well as the dispersion of the dataset. Moreover, in order to assess the diagnostic accuracy of SWE in recognizing thyroid and parathyroid tissues, the receiver operating characteristic (ROC) curves were used, and an optimal threshold value was established for distinguishing between different pathologies in terms of elastography quantitative parameters. Statistical significance was indicated by a p-value threshold of 0.05.

## 3 Results

### 3.1 Group characteristics

In the subsequent **Table 1**, we detail the demographic distribution of the 218 patients included in the analysis, with diffuse thyroid disease and hyperthyroidism confirmed by biochemical assessment and controls; the patients are distributed across different pathology subgroups. In the iatrogenic group, 3 patients were taking amiodarone, 2 patients were administered immune checkpoint inhibitors (1 with Durvalumab for non-small cell lung carcinoma and 1 with ipilimumab for malignant melanoma) and 1 patient received radiation therapy in the past 3 months.

**Table 1:** Demographic Distribution of Subgroups.

		Controls	SAT	GD	Iatrogenic	Painless	Hashi-toxicosis
Number of patients		50	64	87	6	5	6
Gender	Males	11	18	24	4	1	0
	Females	39	46	63	2	4	6
Age		41.9 ± 6.9	42.5 ± 9.1	40 ± 7.2	71.1 ± 4.1	40.2 ± 14.1	43.8 ± 13.5
TSH (mIU/L)		2.2 (2-3.5)	0.02 (0.01-0.09)	0.004 (0.001-0.02)	0.006 (0.001-0.01)	0.03 (0.001-0.12)	0.02 (0.02-0.03)
FT4 (ng/dl)		1.3 (1-1.5)	3.8 (1.8-4.7)	4.1 (1.9-5.8)	4.0 (3.5-5.9)	2.4 (1.9-3.2)	2.7 (1.8-3.5)
FT3 (pg/ml)		3.1 (2.4-4.0)	4.6 (2.9-6.8)	7.5 (5.8-8.8)	6.1 (5.2-7)	5.4 (4-7.1)	5.2 (4.1-7)
ATPO (number of positives)		0 (0%)	5 (7.8%)	38 (43.7%)	1 (16.7%)	1 (20%)	5 (83.3%)
ATG (number of positives)		0 (0%)	2 (3.1%)	15 (17%)	0 (0%)	1 (20%)	2 (33%)
TRAb (number of positives)		0 (0%)	0 (0%)	87 (100%)	0 (0%)	0 (0%)	0 (0%)

SAT, subacute thyroiditis; GD, Graves’ disease; TSH, thyroid stimulating hormone; FT4, free thyroxine; FT3, free triiodothyronine; ATPO, anti-thyroid peroxidase antibodies; ATG, anti-thyroglobulin antibodies; TRAb, Thyroid receptor antibody.

### 3.2 Ultrasound parameters

**Table 2** provides a detailed comparison of the ultrasound-based thyroid parameters across the six distinct subgroups. The results of a one-way ANOVA conducted for each parameter are also presented, helping to discern the significance of observed differences. The p-value (<0.001) for all parameters indicates a significant difference among the subgroups for each respective measurement.

**Table 2:** Thyroid parameters according to disease-type subgroups.

	Controls	SAT	GD	Iatro-genic	Pain-less	Hashi-toxicosis	P (ANOVA)
Thyroid Volume (ml)	15 (12-18.1)	24.3 (21.3-26.8)	24.4 (20-31.8)	19.9 (17.7-24.3)	25.5 (23.2-28.3)	14.5 (10-18)	<0.001
Hypoecho-genicity	3/50 6%	64/64 100%	81/87 93.1%	6/6 100%	4/5 80%	4/6 66.6%	<0.001
Color Doppler	1 (0-1)	1 (0-1)	2 (2-3)	1	1 (0-1)	2	<0.001
PSV (cm/sec)	11.8 (11.3-13.4)	8.6 (7-10.7)	42.4 (36.9-48)	9.4 (7.8-11.5)	14.8 (14.7-15.4)	19.2 (17.8-20.6)	<0.001
Emean-SWE (kPa)	12.3 (11.1-13.8)	111.6 (95.2-118.2)	15.5 (11.6-17.4)	12.9 (10.5-17.8)	28.9 (26.6-34.1)	29.3 (21.5-34.2)	<0.001
Emin-SWE (kPa)	9.6 (8.6-11.3)	80.8 (69.7-90.2)	10.9 (8.9-12.5)	9.2 (7.3-12.5)	19.1 (18.8-22)	19.2 (18.3-26.5)	<0.001
Emax-SWE (kPa)	15.3 (13.2-19)	128.9 (109.2-137.9)	20.3 (17.9-22.7)	12.9 (13.8-21.2)	39.6 (34.2-41.7)	36.4 (26.9-44.9)	<0.001

PSV, peak systolic velocity; Emean, mean elasticity index; SWE, shear-wave elastography; Emin, minimum elasticity index; Emax, maximum elasticity index; GD, Graves’ disease.

In terms of subgroups for Emean, the SAT group has much higher median values than the other groups. No statistically significant difference was detected between: controls and GD, painless and iatrogenic causes; GD and iatrogenic disease; hashitoxicosis and painless thyroiditis.

In terms of subgroups for Emin, SAT also has the highest elastography value, significantly differing from the other subgroups. No statistically significant difference was seen between: painless thyroiditis and hashitoxicosis, iatrogenia and GD; hashitoxicosis and controls; GD and controls. As for the Emax, once again, the SAT subgroup shows the highest maximum elastography measurement, significantly outlying the other groups. There was no statistically significant difference only between: painless thyroiditis and hashitoxicosis; iatrogenic events and GD and controls.

For thyroid volume, both GD and Painless subgroups exhibit a higher thyroid volume, while the Hashitoxicosis subgroup shows the lowest. The differences between the groups were significant. Taken in pairs, there was statistical significance regarding the differences only between: controls and SAT, painless thyroiditis, iatrogenic causes and GD and between hashitoxicosis and SAT, iatrogenic thyroiditis and GD.

For Color Doppler Schultz scale assessment, there were significant differences between GD and all other subgroups except from the small Hashitoxicosis group. No significant differences were detected between the destructive thyroiditis subgroups (subacute, iatrogenic, painless thyroiditis). The PSV values were the highest among the GD subgroup, significantly different from all the other groups. Statistical significance was also found between SAT and controls and hashitoxicosis and between hashitoxicosis and controls.

### 3.3 Step I: B-mode ± color doppler

**Figure 1:**
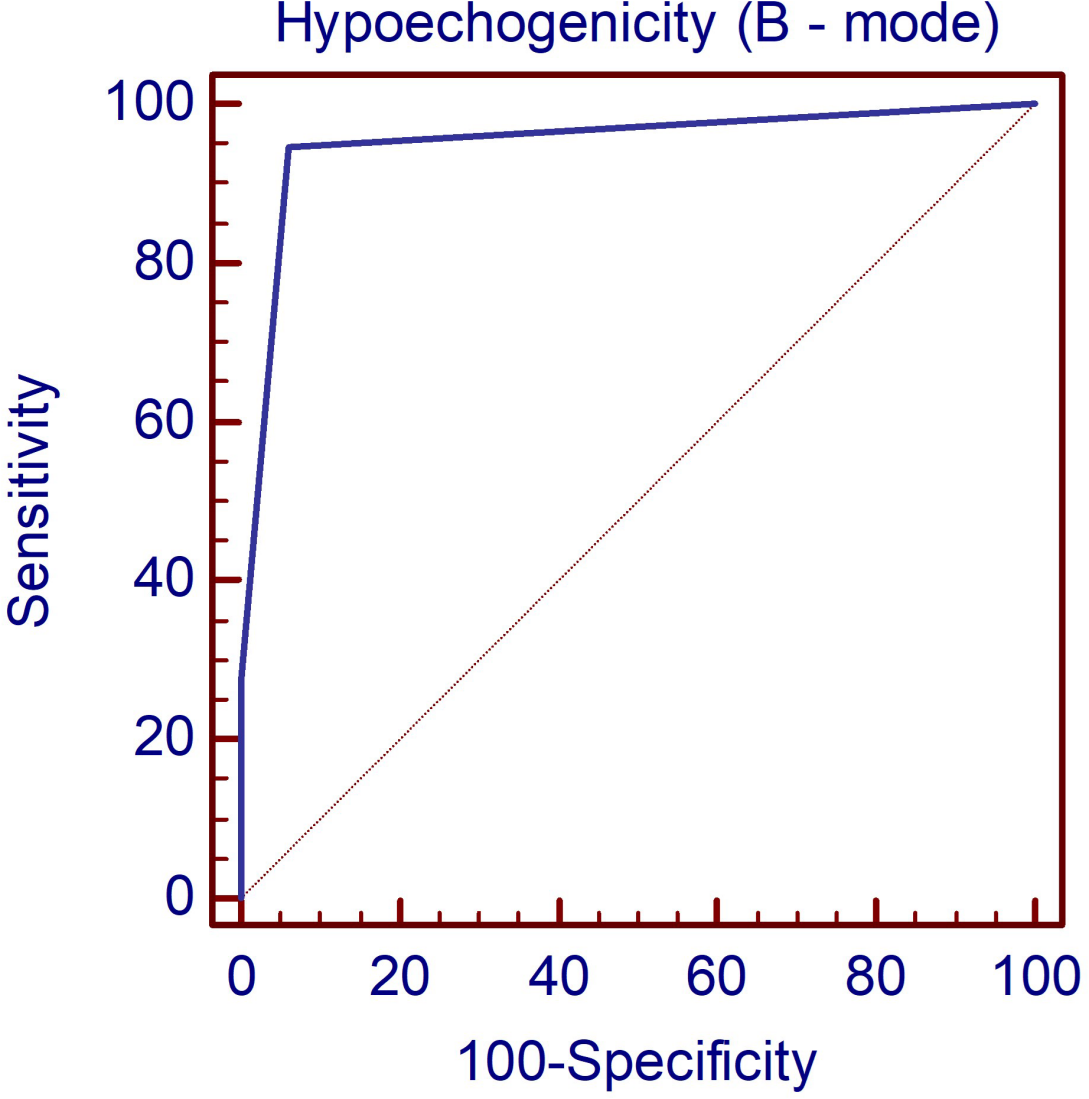
The AUC illustrating the diagnostic performance of the B-mode parameter hypoechogenicity for detecting DTD. DTD, diffuse thyroid disease.

In the first step of evaluation, the B-mode hypoechogenicity was assessed and a value for the AUC of 0.951 was found as shown in **Figure 1**, with sensitivity of 94.6% and specificity of 94%, when the presence of hypoechogenicity was considered predictive for the presence of diffuse thyroid disease of any type, regardless of the intensity of the hypoechogenicity.

**Figure 2:**
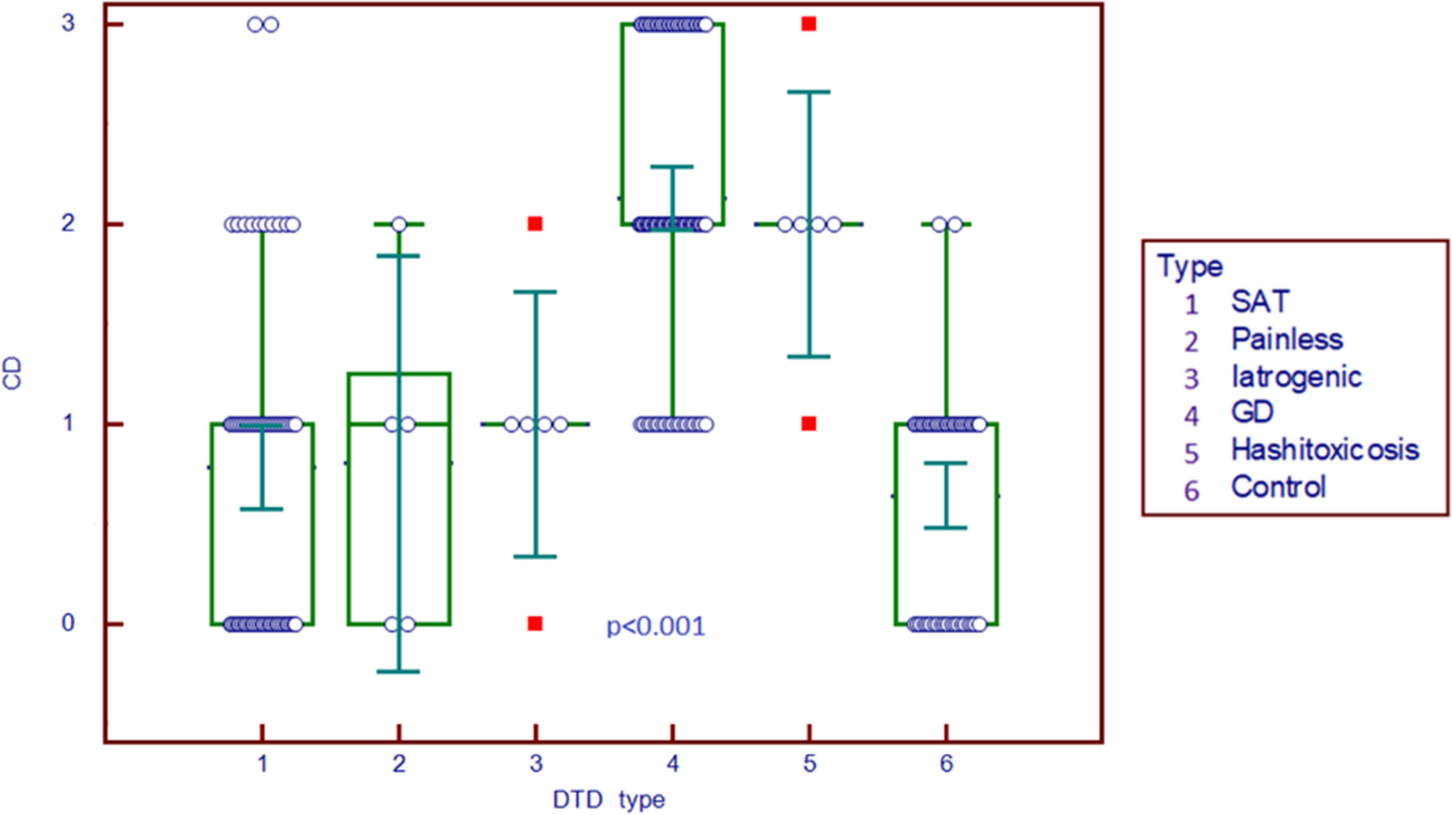
Comparison graph for Color Doppler scale in pathology subgroups and controls. CD, Color Doppler; DTD, diffuse thyroid disease; SAT, subacute thyroiditis; GD, Graves’ disease.

The differences across different types of thyroid pathologies in terms of Color Doppler graded by the Schulz scale (0–3) are displayed in **Figure 2**. Color Doppler scale was increased in GD and HT compared to all other subgroups (p<0.001).

### 3.4 Step II: Spectral doppler

**Figure 3:**
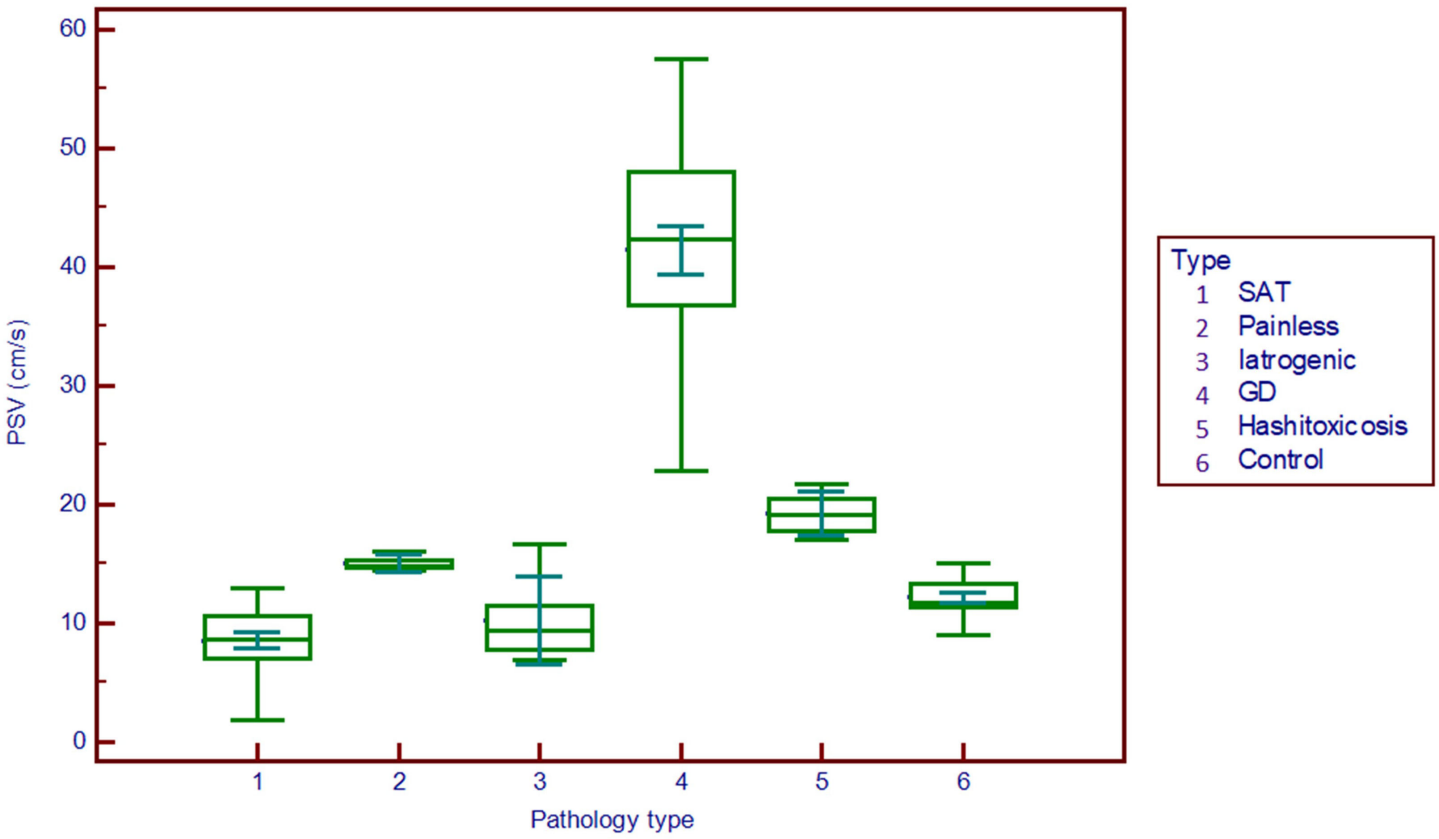
Comparison graph for peak systolic velocity values in pathology subgroups and controls. PSV, peak systolic velocity; SAT, subacute thyroiditis; GD, Graves’ disease.

The peak systolic velocity measurements seem to be able to differentiate GD (median 42.4 cm/s) from all other forms of DTD (median 9.2 cm/s) even from HT (median 19.2 cm/s) and also from controls (median 11.8 cm/s) (p<0.001), as shown in **Figure 3**.

### 3.5 Step III: Elastography parameters

**Figure 4:**
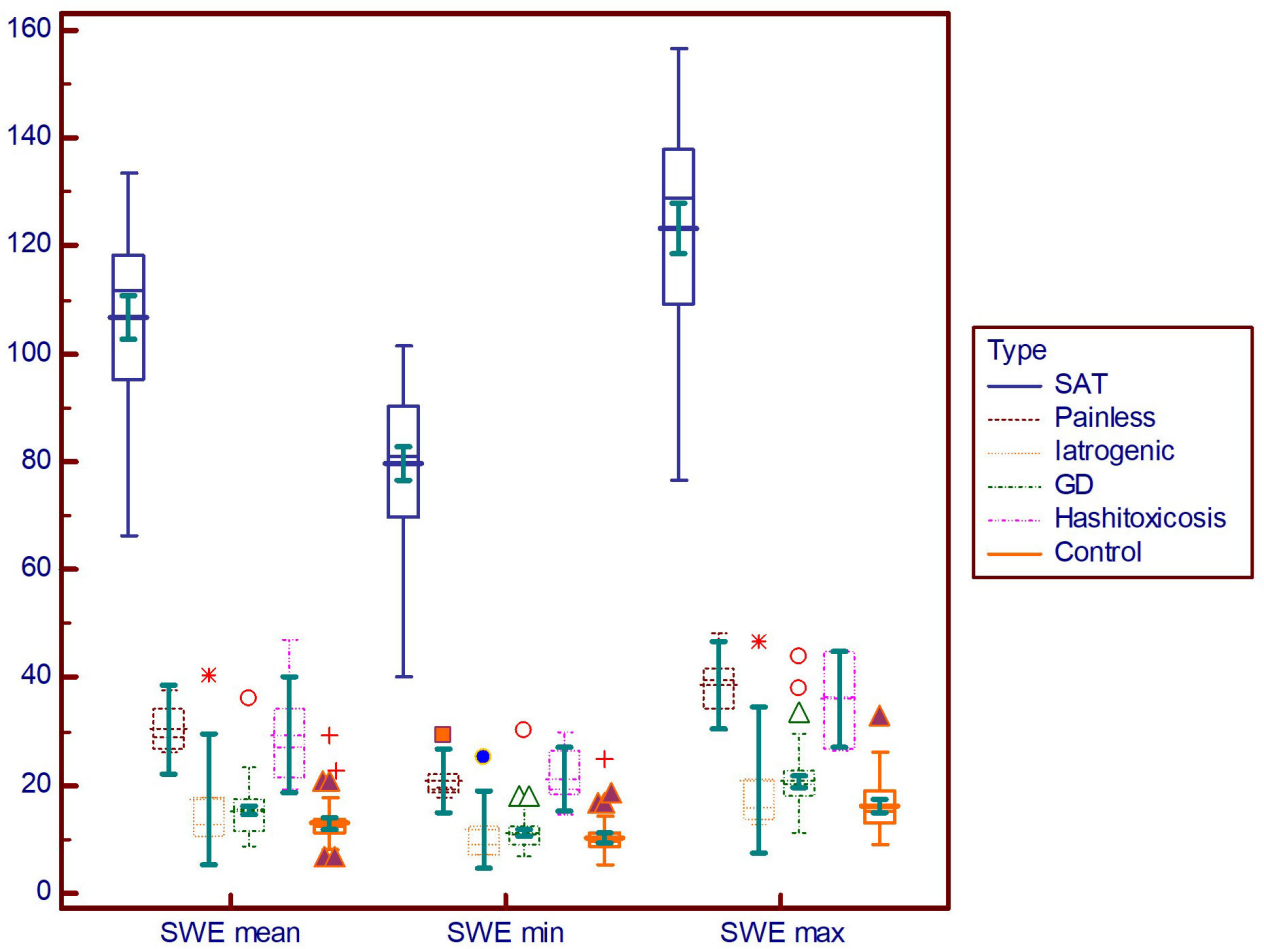
Comparison graph for shear-wave elastography values in pathology subgroups. SWE mean, mean elasticity index; SWE min, minimum elasticity index; SWE max, maximum elasticity index; PSV, peak systolic velocity; SAT, subacute thyroiditis; GD, Graves’ disease.

**Figure 4** displays the differences in different DTD subgroups and controls for elasticity parameters (E mean; Emin; Emax). The SAT subgroup displays significantly higher values for all elasticity parameters compared to all other subgroups (p<0.001).

### 3.6 DTD differential diagnosis: SAT versus non-SAT hyperthyroid DTD

**Figure 5:**
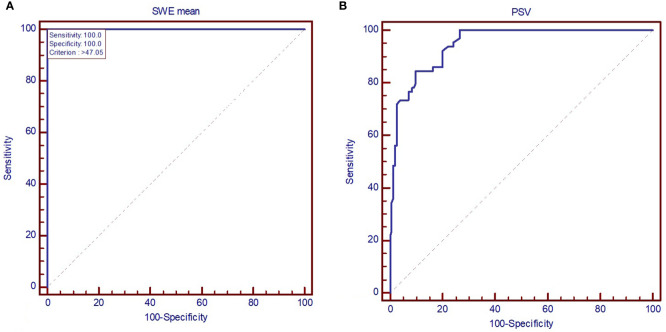
AUC displaying the performance of **(A)** mean elastic index measured by shear-wave elastography and **(B)** peak systolic velocity in diagnosing subacute thyroiditis (SAT). SWE mean, mean elasticity index; PSV, peak systolic velocity.

The AUC value depicts excellent diagnostic performance of 2D SWE in detecting SAT from other forms of hyperthyroid DTD at onset. **Figure 5** shows the AUC for a) 2D SWE and b) PSV in detecting SAT from DTD in our group.

All the US-based parameters are detailed in **Table 3** for differentiating SAT from other forms of hyperthyroid DTD.

**Table 3:** Ultrasound parameters in SAT versus non-SAT hyperthyroid DTD.

	AUC	Cut-off	Se	Sp	p
2B					
Hypoechogenicity	0.539	>0	98.4%	25.9%	<0.0001
TV	0.511	>21.2 ml	79.7%	45.2%	<0.0001
CD					
Schulz scale	0.832	≤1	81.3%	72.1%	<0.0001
PSV					
PSV	0.977	≤11 cm/s	84.4%	96.2%	<0.0001
2D SWE					
Emean	1	>47 kPa	100%	100%	<0.0001
Emax	1	>48 kPa	100%	100%	<0.0001
Emin	1	>30 kPa	100%	100%	<0.0001

AUC, area under the curve; 2B, B-mode ultrasound; CD, Color Doppler; PSV, peak systolic velocity; 2D SWE, bidimensional shear-wave elastography; Emean, mean elasticity index; SWE, shear-wave elastography; Emax, maximum elasticity index; Emin, minimum elasticity index.

**Figure 6:**
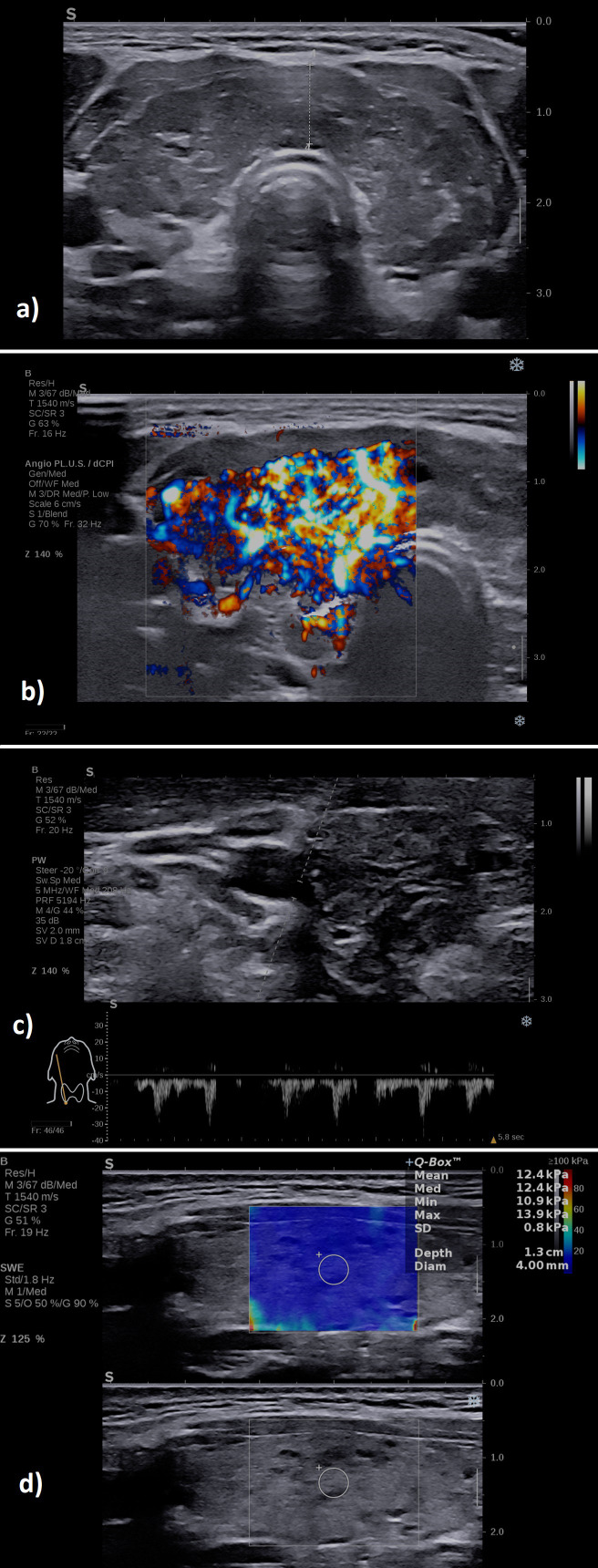
A case of a patient with subacute thyroiditis: **(A)** B-mode examination of the right thyroid lobe in sagittal plane: diffusely hypoechoic; **(B)** with increased vascularity grade II Color Doppler, image is obtained using the fine-flow mode called ANGIOPLUS on the equipment used in this study; **(C)** spectral Doppler lower PSV in the thyroid artery; **(D)** increased mean elasticity index measured in 2D SWE 109.7 kPa.

**Figure 7:**
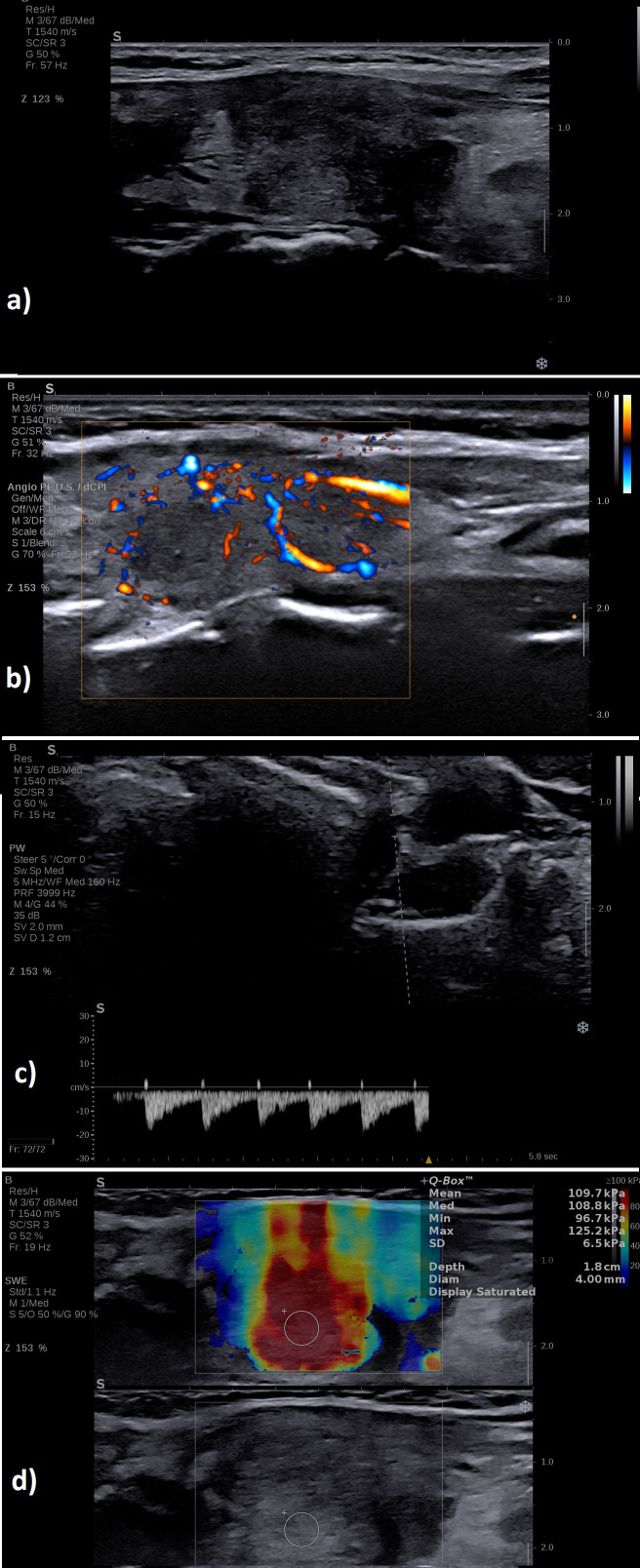
A case of a patient with Graves’ disease: **(A)** B-mode examination of the entire thyroid in transverse plane: diffusely enlarged with enlarged isthmus of 9 mm, diffusely hypoechoic; **(B)** with increased vascularity grade III Color Doppler, image is obtained using the fine-flow mode called ANGIOPLUS on the equipment used in this study; **(C)** spectral Doppler increased PSV in the thyroid artery; **(D)** normal mean elasticity index measured in 2D SWE 12 kPa.

A typical case of SAT in the hyperthyroid phase is illustrated in **Figure 6** in the multiparametric US-based approach. **Figure 7** displays a multiparamerric US imaging in a case of Graves disease

### 3.7 DTD differential diagnosis: GD form destructive forms of DTD

All the US-based parameters are detailed in **Table 4** for differentiating GD from destructive DTD.

**Table 4:** Ultrasound parameters in GD versus other hyperthyroid DTD.

	AUC	Cut-off	Se	Sp	p
2B					
Hypoechogenicity	0.550	≥2	35.7%	84%	<0.0001
TV	0.545	>29 ml	33.4%	92.6%	<0.0001
CD					
Schulz scale	0.841	≥2	78.2%	76.6%	<0.0001
PSV					
PSV	1	>22 cm/s	100%	100%	<0.0001
2D SWE					
Emean	0.953	≤24 kPa	98.9%	91.3%	<0.0001
Emax	0.945	≤30 kPa	96.6%	91.4%	<0.0001
Emin	0.951	≤18 kPa	98.9%	91.4%	<0.0001

AUC, area under the curve; 2B, B-mode ultrasound; CD, Color Doppler; PSV, peak systolic velocity; 2D SWE, bidimensional shear-wave elastography; Emean, mean elasticity index; SWE, shear-wave elastography; Emax, maximum elasticity index; Emin, minimum elasticity index.

The parameters in **Tables 3** and **4** relate only to the differential diagnosis between the different types of hyperthyroid DTD analyzed in this study.

### 3.8 Multiparametric ultrasound algorithm

**Figure 8:**
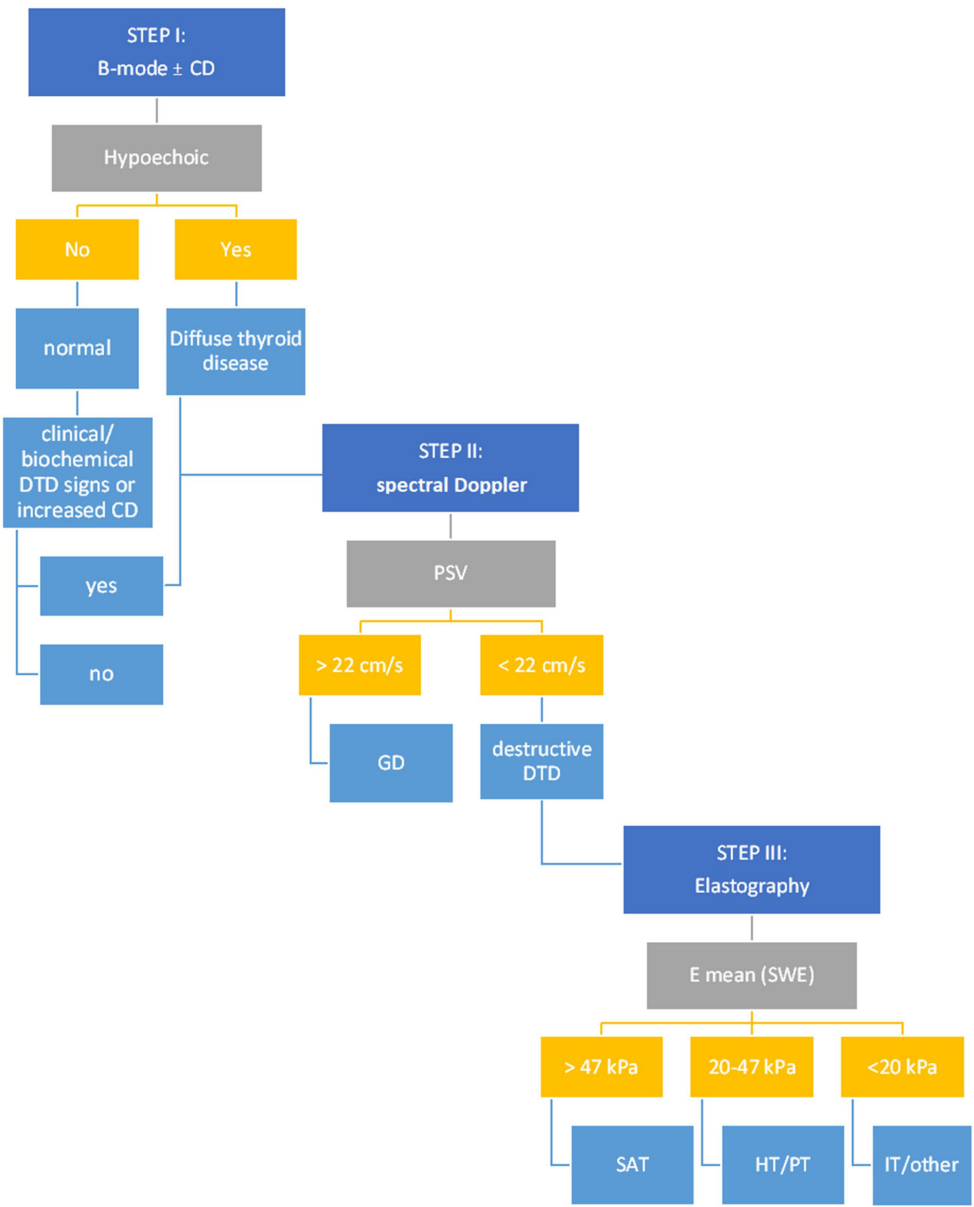
The multiparametric ultrasound algorithm for DTD evaluation. CD, Color-Doppler; DTD, diffuse thyroid disease; PSV, peak systolic velocity; GD, Graves’ disease; E mean, mean elasticity index; SWE, shear wave elastography; SAT, subacute thyroiditis; HT, Hashitoxicosis; PT, painless thyroiditis; IT, iatrogenic thyroiditis.

After the step-by-step evaluation and the results provided so far, a stepwise algorithm was proposed, detailed in **Figure 8**.

**Figure 9:**
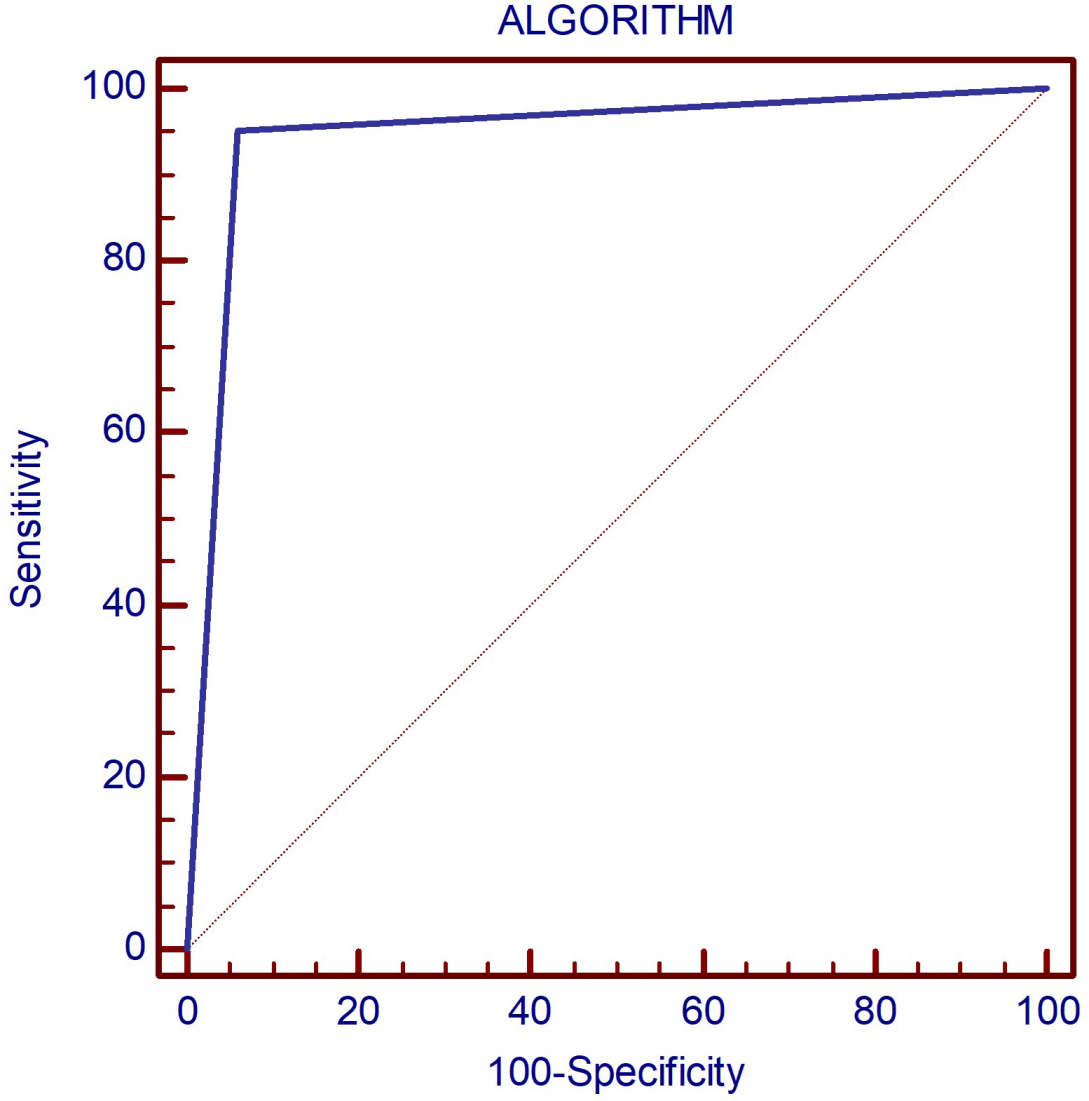
The AUC displaying the diagnostic performance of the proposed algorithm for correctly identifying DTD subtype as well as normal thyroid on multiparametric ultrasound.

The overall accuracy rates for the proposed algorithm are excellent (**Figure 9**): AUC=0.946; Se=95.2%; Sp=94%. The algorithm misclassified 3/50 controls and 8/168 DTD cases. In the control group, three cases were initially misclassified as DTD due to the presence of hypoechogenicity in 2B ultrasound evaluation. One of these cases also exhibited an increased Color Doppler pattern, which may further contribute to the misclassification. Within the DTD group, there were misclassifications at the subtype level. Specifically, cases were initially classified into non-SAT destructive forms of thyroiditis, including iatrogenic, painless, and hashitoxicosis. While these misclassifications occurred, it’s important to note that all of these cases were correctly identified as destructive forms of thyroiditis, thus the correct therapeutic approaches were still followed for these cases. The heterogeneity within the iatrogenic subgroup and the smaller number of cases in some subgroups may have influenced these misclassifications.

## 4 Discussions

In our research, B-mode ultrasound emerged as a valuable diagnostic tool for distinguishing normal thyroid tissue from diffusely affected thyroid parenchyma. However, it’s important to underscore that our study exclusively assessed patients presenting with hyperthyroid DTDs and did not encompass those with euthyroid or hypothyroid DTDs.

Further, it was demonstrated in this study that PSV serves as a reliable diagnostic tool for distinguishing Graves’ Disease (GD) with a median value of 42.4 cm/s from other forms of Diffuse Thyroid Diseases (DTD) which had a median of 9.2 cm/s. Remarkably, the diagnostic accuracy in our study cohort was shown by AUC of 1, indicating excellent performance. The cut-off threshold identified in our study is more conservative than those previously documented in the literature.

It is known that individuals with Graves’ disease exhibit markedly elevated FT3, FT4, TRAb levels, and thyroid volume in comparison to those diagnosed with thyroiditis. Similarly to our findings, other authors also found that the average PSV for Graves’ disease surpassed that of thyroiditis. The PSV threshold values that have been identified so far in the literature, of 40-50 cm/sec (30, 33, 34) provided a differential diagnostic ability with an accuracy of 88%, comparable with the reference differential method, thyroid scintigraphy (28). Both GD and initial phases of HT have higher PSV values compared with normal controls (30), significantly higher in overproduction cases compared with destructive processes, regardless of the background, subacute or autoimmune. No major differences have been detected between different types of destructive thyroiditis, such as SAT versus postpartum/painless thyroiditis, suggesting that all destructive thyroids show low flow pattern (41, 42). In a study by Donkol et al. (30) on the differential diagnosis of GD and HT, Doppler US demonstrated a sensitivity of 88.9%, a specificity of 87.5%, a positive predictive value of 94.1%, a negative predictive value of 77.8%, and a diagnostic accuracy of 88.5% compared the reference standard of ^99m^Tc scintigraphy. Similarly, in a study by Hari Kumar et al. (43), Doppler US had a sensitivity of 96% and specificity of 95% for the differential diagnosis of thyrotoxicosis in reference to ^99m^Tc scintigraphy. Moreover, PSV demonstrated a better AUROC area than AUROC of both TRAB values, and the triiodothyronine (T3) to thyroxine (T4) ratio (34), two of the most used biochemical criteria for GD confirmation. There is generally no substantial difference between PSV measurements made on the superior or inferior thyroid arteries or on the inferior thyroidal arteries of either side. In one particular study, a mean superior thyroid artery (STA) PSV exceeding 54.3 cm/s and a mean PSV-STA/PSV-common carotid artery ratio beyond 0.4 had strong sensitivity and specificity in identifying Graves’ disease. These metrics can therefore be valuable in distinguishing between the two conditions (34, 42).

Elastography is another very important US-based tool for thyroid evaluation. In our study it proved particularly helpful in detecting SAT. All elasticity parameters for the SAT subgroup were notably elevated compared with all other subgroups, with the difference being statistically significant (p<0.001) and an AUC of 1 in distinguishing SAT from other forms of DTD, thus establishing its place in the algorithm we provided. When looking into the different types of autoimmune disease, the mean values described so far by a number of studies show greater stiffness for HT compared with the mean values for GD (39, 44–46). The data are discordant since some authors described the differences in stiffness as significant (46) while others did not (39, 44, 45). When analyzing other types of thyroiditis, major differences are described between the stiffness observed in subacute thyroiditis (SAT), with mean elasticity values for 2D SWE over 150 kPa, regardless of the viral-type background and the stiffness of autoimmune types of diseases such as GD or Hashimoto, with mean elasticity values of 25 kPa (47, 48). The AUC of SWE was 0.549 and 0.989, respectively, for HT from GD and SAT, while the fT3/fT4 ratio and SWE had AUCs of 0.975 and 0.713 for separating GD from SAT. Strain elastography (SE) also showed similar results. In terms of sensitivity, specificity, and area under the curve (AUC), the cut-off points for strain elastography in patients with GD, HT, and SAT to the CG were 2.69 (sensitivity 92%, specificity 90%, AUC 0.983; 95% CI), 2.18 (sensitivity 100%, specificity 85%, AUC 0.898), and 5.54 (sensitivity 100%, specificity 100%, AUC 1.000; 95% CI), respectively. Strain ratios (SR) of all patients with HT and GD to SAT were cut off at 14.79 (sensitivity 80%, specificity 85%, AUC 0.869; 95% CI) (49).

In our proposed stepwise algorithm, patients are first evaluated with B-mode ultrasound. Those exhibiting either mild or intense hypoechogenicity within the thyroid parenchyma or a grade 2-3 Schultz evaluation in Color Doppler, or those with normal echogenicity but concomitant biochemical or clinical indications of Diffuse Thyroid Diseases (DTD) are identified as suitable candidates for the multiparametric US evaluation. This subsequently leads them to the second step of the assessment.

In step 2, a PSV exceeding 22 cm/s is highly suggestive of Graves’ Disease (GD). In all other cases, the evaluation must advance to step 3: the 2D-SWE. If the mean elasticity (Emean) exceeds 47 kPa, SAT is strongly suspected. Conversely, autoimmune-mediated forms such as HT and/or PT typically present Emean values ranging between 20-47 kPa. Iatrogenic manifestations are indicated by Emean values that are typically less than 20 kPa.

Color Doppler evaluation was optional and was not considered as a separate step in our algorithm, as its overall diagnostic performance was inferior to other methods. However, when CD was increased in step I with no other signs of DTD, further evaluation is necessary for excluding DTD; it may also be used to reconfirm the presence of DTD in conjunction with the presence of hyopechogenicity and to reconfirm the presence of GD together with increased PSV.

Our imaging approach offers several advantages compared to conventional clinical models. Clinical assessments often rely on symptoms and signs that can overlap across different thyroid conditions, leading to diagnostic challenges. Furthermore, the evolving landscape of thyroid disease presentation, including cases with atypical features or post-COVID-19 subacute thyroiditis, has rendered clinical examination alone less reliable. Our algorithm, based on objective imaging data, provides precise information on thyroid texture, vascular patterns, and elastography measurements. By doing so, it enhances diagnostic accuracy, aids in distinguishing various hyperthyroid conditions, and ensures a more tailored therapeutic approach.

Our algorithm has successfully fulfilled its primary objective, which was to distinguish between hyperthyroidism cases based on the required therapeutic approach. Specifically, it effectively differentiated cases where antithyroid drugs were warranted for hyperfunction from cases necessitating specific treatments for destructive, self-limited forms of thyroid disease. This accomplishment underscores the clinical relevance and utility of our algorithm in guiding quick and appropriate therapeutic interventions for patients with varying thyroid pathologies with hyperthyroidism at onset.

## 5 Conclusions

Integrating insights from gray scale US, color and spectral Doppler, and elastography, we can obtain critical information on diffuse thyroid diseases. Moreover, the structured stepwise algorithm reduces both expenses and the duration of investigations providing a reliable means of diagnosis and therapeutic approach in daily clinical practice.

## Data availability statement

The original contributions presented in the study are included in the article/**Supplementary Material**, further inquiries can be directed to the corresponding author.

## Ethics statement

The studies involving humans were approved by Comisia Locala De Etica Pentru Cercetere Ştiinţifică A Spitalului Clinic Judeţean De Urgenţă ,,Piu Brînzeu” Timişoara. The studies were conducted in accordance with the local legislation and institutional requirements. The participants provided their written informed consent to participate in this study.

## Author contributions

DS: Conceptualization, Formal analysis, Investigation, Project administration, Resources, Visualization, Writing – original draft, Writing – review & editing. AB: Data curation, Investigation, Methodology, Project administration, Writing – original draft, Writing – review & editing. LM-L: Methodology, Supervision, Validation, Writing – review & editing. CP: Conceptualization, Supervision, Validation, Writing – review & editing.
