# Supplementary figures and images for: Multiparametric ultrasound-based assessment of overt hyperthyroid diffuse thyroid disease
Source: Front Endocrinol (Lausanne). 2023 Dec 18;14:1300447. doi: 10.3389/fendo.2023.1300447 (PMC10764279; doi:10.3389/fendo.2023.1300447)

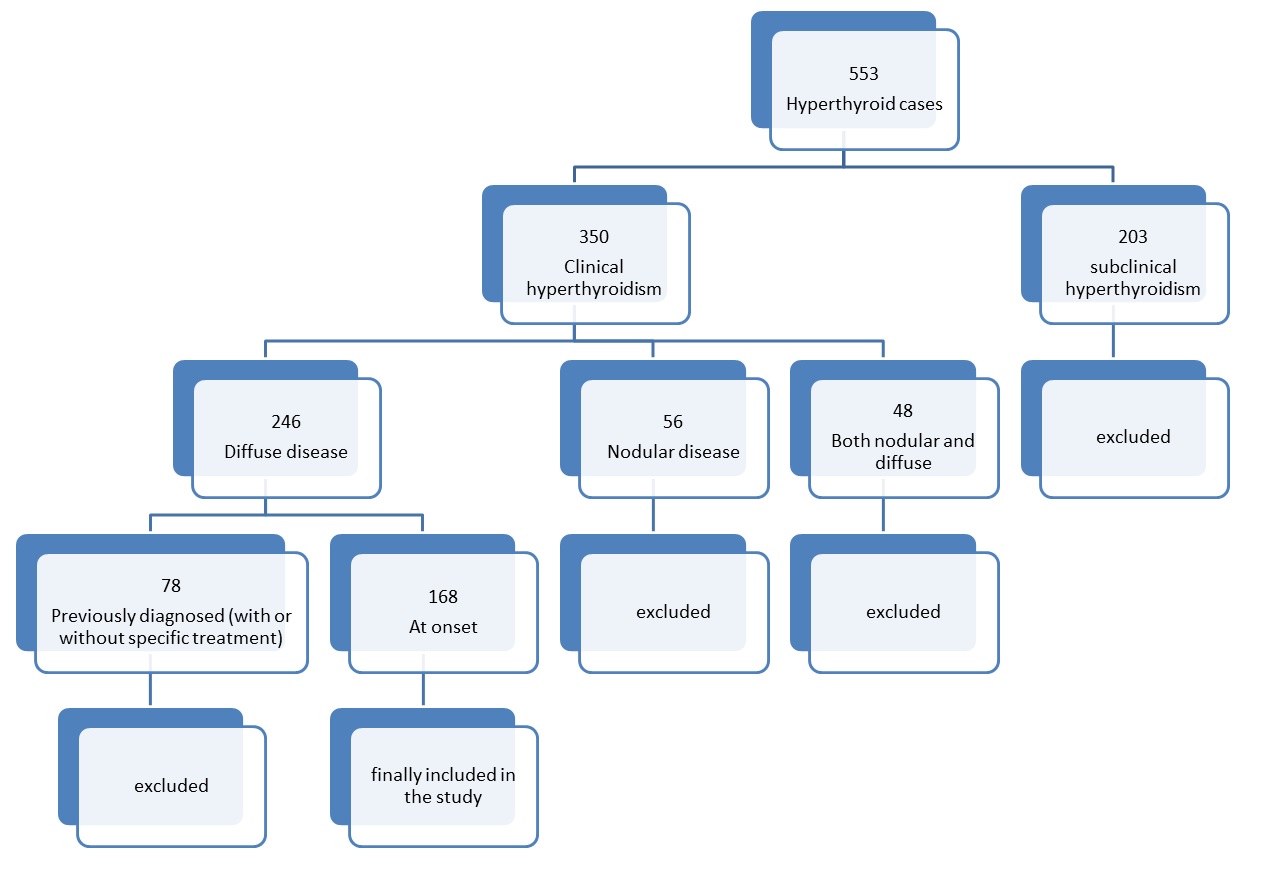

Supplement: Supplementary file 1 [file Image_1.jpeg]
